# Social Perception of Non-Binary Individuals

**DOI:** 10.1007/s10508-021-02234-y

**Published:** 2022-04-25

**Authors:** Karolina Hansen, Katarzyna Żółtak

**Affiliations:** Faculty of Psychology, University of Warsaw, Stawki 5/7, 00-183 Warsaw, Poland

**Keywords:** Non-binary, LGBTQ+, Polish language, Gender-neutral language, Social perception, Transgender

## Abstract

People can express their identity in different ways, one of which is through language. Non-binary individuals often speak in a gender-neutral way and use specific language forms. Language use not only reveals their identity but also can shape how others perceive them. The present study’s purpose was to analyze how non-binary people are perceived through the language they use. The research was conducted in Polish, a language that is especially challenging for non-binary individuals because it has many gender markers. To avoid using gendered forms, they often use a specific form of passive voice. In an experiment, participants (*N* = 130, 102 women, 28 men) read a gendered (feminine or masculine) text and a gender-neutral text with passive voice. Most gave a masculine name to the person in the neutral text, but addressed them in a gender-neutral way when asked to react to them in presented scenarios. The gender-neutral text was evaluated as being less comprehensible than the gendered texts, and the non-binary person was rated less competent and colder than a man or a woman and was less socially accepted. Furthermore, the negative evaluation of non-binary people seemed to be attributable to unfamiliarity with gender-neutral language and its lower comprehensibility. More research is needed to understand these perceptions better and to be able to prevent their potential negative consequences.

**Supplementary Information:**

The online version contains supplementary material available at 10.1007/s10508-021-02234-y.

## Introduction

One important way to express one’s gender identity is through language. Within the LGBTQ+ community: non-binary people. The term non-binary can be described as an umbrella term for individuals who do not identify as male or female (Barker, 2017), encompassing people with various identities and experiences (Titman, 2014). These people “do not have a gender,” “feel partially male or female,” “are both masculine and feminine,” “are located somewhere between male and female,” “have an additional […] gender,” or “move between genders” (Barker, 2017, p. 27). More specifically, the term non-binary refers more to how a person identifies themselves than simply their physicality (Richards et al., 2017). Non-binary English-speaking individuals or others speaking about such individuals often use the singular they to be gender neutral (Wong, 2017). However, the real struggle begins when one speaks a gendered language. In this kind of language, gender is reflected not only in personal pronouns but also in nouns, adjectives, and adverbs (Hord, 2016). In Polish, which is a heavily gendered language, the gender is visible in all mentioned cases––and even in verbs in many tenses. It is hard to express one’s non-binary gender identity through the Polish language, but Polish non-binary individuals have found a way to do so: Some of them use a specific form of passive voice. This article’s purpose is to study the social perception of non-binary individuals and their language.

### Importance of Gender-Neutral Language

The language of non-binary people is a matter of inclusiveness. If one does not have a language with which to express their identity, they feel invisible (Ivy, 2016). Therefore, non-binary people use existing language forms, change their meaning, or create new forms (Wong, 2017). Even when non-binary individuals have a language to describe themselves, they still often are misgendered (given an incorrect gender through language) by others (Hord, 2016). This happens because “gender is not always visible” (Wong, 2017, p. 17), and for strangers, it may be hard to determine a person’s gender identity. This can lead to discomfort and distress among non-binary individuals, as well as among those who realize that they misgendered others unintentionally (Barker, 2017). When a non-binary person asks others to use different pronouns and language when referring to them, they are *coming out* and are at risk of being misunderstood or rejected. An example of this can be found in what Respondent 89 said in a study by Hord (2016, p. 21): “The neo-pronouns I use better express [my gender identity], but besides not always being accessible, they’re not well-known, and I’d have a nearly impossible time getting people in my daily life (not friends who understand) to use them. I usually have to fight just to get the barest amount of gender-neutral language used for me, let alone gender-expressive language.”

Non-binary people struggle often when speaking in gendered language and have an especially hard time displaying their self-identification through such language. As another person explains: “[In] German, I struggle a lot with language, and [I am] often very unhappy with the situation of [the lack of] German gender-neutral language. I lack usable and easy-to-learn/apply pronouns and descriptions of myself. That the language is very gendered is a big problem in my life” (Hord, 2016, p. 21).

### Non-Binary Polish Language

Language used by non-binary people varies from individual to individual, with some using male, female, or neutral pronouns, sometimes in different combinations depending on the situation, interlocutor, etc. Non-binary language also varies often depending on the language used and linguistic possibilities available in that language. Personal pronouns, nouns, and even adjectives and past-tense verbs in Polish require deciding between using a masculine or a feminine version of a word (Hord, 2016).

When it comes to personal pronouns, some Polish non-binary people use or ask others to refer to them with *ono* or *onu*, which are a real (*ono*) and an invented (*onu*) neutral version of *on* (he) and *ona* (she) that can be considered equivalents of the singular *they* (Angry Trans, 2018). *Ono* is a neutral Polish pronoun that is used when talking about animals and some objects but also can be used to describe a baby or a child (Bąk, 1984). *Onu* is a pronoun invented by Jacek Dukaj, a Polish author who introduced it in his book *Perfekcyjna Niedoskonałość* (Dukaj, 2004). It then started to be used by or to refer to non-binary people (Helena, 2018). Some non-binary people simply use the noun person (Helena, 2018). Generally, instead of using a gendered noun to describe themselves, for example, saying *jestem psychologiem*_*mas*_ or *jestem psycholożką*_*fem*_ (I am a psychologist), a non-binary person may say *jestem osobą psychologa* (I am a psychologist person) or *jestem osobą profesji psychologicznej* (I am a person of psychological profession; Helena, 2018). Regarding adjectives, in Polish, to say *I am happy*, the adjective *happy* needs to have a masculine or a feminine ending (*jestem wesoły*_*mas*_ or *jestem wesoła*_*fem*_). To avoid this, one can use an adverb (*jest mi wesoło*).

Past-tense verbs also are gendered and often end with –*em* for masculine forms and –*am* for feminine ones. Some non-binary people use –*um* or –*om* endings as an alternative (Helena, 2018). An interesting construction that some non-binary individuals use in this case is a specific form of passive voice. Instead of saying *czytałam książkę* (*I was reading*_*fem*_* a book*) or *czytałem książkę* (*I was reading*_*mas*_* a book*), they say *czytało mi się książkę*. This is not a standard passive voice, like *the book was read by me* (*książka była czytana przeze mnie*), but rather reflects the English *I’ve got my hair cut*, or in this case, *I’ve got my book read*. In Polish, it emphasizes the activity, rather than the person, and is gender neutral. Another example is the sentence *Szłam ulicą* (*I was walking*_*fem*_* down the street*) or *Szedłem ulicą* (*I was walking*_*mas*_* down the street*). Instead of using the gendered forms, a non-binary person could say *Szło mi się ulicą,* which is gender neutral.

Non-binary people choose the language that they, themselves, want to use, but how should others address them? In Polish, some non-binary individuals prefer that strangers address them directly using *you* instead of the more common and polite, but gender marked, *Sir/Madam* (Petriczko, 2015). For example, instead of asking C*zy chciał(a)by Pan/i coś do picia?* (*Would*_*mas/fem*_* you Sir/Madam like something to drink?*), one can say *Czy chcesz coś do picia?* (*Would you like something to drink?*).

### The Current Research

The present study’s focus was on how others perceive non-binary individuals through the language they use. While gender-fair language focuses on personal pronouns and nouns, especially professional names, we focused on omnipresent verbs (Sczesny et al., 2016; Tavits & Perez, 2019). There were various reasons why we chose verbs and gender-neutral passive voice that non-binary people use: Verbs are prevalent and impossible to avoid, and both non-binary and other people use them when talking about non-binary people. We focused only on one part of speech to determine precisely what influenced how non-binary people were perceived.

In terms of evaluations of non-binary people, we examined the main and universal dimensions of social perception: warmth and competence (Cuddy et al., 2008; Fiske et al., 2007). However, in studies on gender-fair language, these often are combined into a general evaluation, which we also did (Formanowicz et al., 2013).

To the best of our knowledge, there are no studies on how people perceive non-binary Polish language, and generally, hardly any research has been done on the topic in other languages. Considering that no study like this has been done before, the current study is exploratory. However, based on some other research, one can have two tentative hypotheses.

First, when compared with standard feminine and masculine language, others may perceive non-binary language and individuals using it negatively. This may be the case because passive voice generally is used rarely in the Polish language, and it is especially rare to use mainly passive voice when speaking. Furthermore, the passive voice that non-binary people use is not a standard passive voice in Polish and might be unfamiliar to many. A person not using a normal, universal language may be viewed as bizarre and evaluated negatively (Tavits & Perez, 2019). Furthermore, research has shown that people use passive voice more frequently when speaking about negative emotions (Kałkus, 2015).

Second, beyond evaluation of the text and the speaker, when it comes to whom people imagine while reading, a large body of research has shown that men are perceived as a norm (Sczesny et al., 2016). Although a consequent use of gender-fair language can in a long run shift people’s language use and attitudes (Tavits & Perez, 2019), in Poland where such efforts are only recent, we expect a standard effect that even when reading gender-neutral texts, more participants imagine men than women (Hodel et al., 2017; Sczesny et al., 2016).

## Method

The present study comprised an online questionnaire with three versions of a written text: masculine, feminine, and gender neutral. Each participant was assigned randomly to receive either a masculine and a gender-neutral text, or a feminine and a gender-neutral text. There were also two versions of each text in terms of the text plot. In both versions, the narrator described their day. In one version, they did so surrounded by friends (Text A), and in the second version, they were in a store and answered a phone (Text B). Therefore, there were four possible versions of the questionnaire. If the participant was assigned randomly to a condition with a masculine/feminine Text A, they were automatically assigned to receive a gender-neutral Text B. In the case of being assigned to a masculine/feminine Text B, the second text was a gender-neutral Text A. After each of the texts, participants answered questions regarding the text and its narrator.

### Participants

The sample was collected using a snowball method. The questionnaire was posted on a private social media profile with a request to share it with others. Altogether, 222 participants clicked the study link, and 133 filled out the entire questionnaire. We excluded one trans-man and two non-binary people from the analyses. Thus, the maximum final sample was *N* = 130 (102 women, 28 men).^1^ Participants, on average, were in their mid-20 s (*M*_age_ = 26.80, *SD* = 10.03), with most studying at a university (41%) or holding a higher education degree (39%). Most lived in a city of more than 500,000 residents (37%), but there were also many from medium-size cities (18%), smaller cities (21%), towns (11%), or villages (13%).

### Procedure and Measures

Participants were informed that the questionnaire comprises three parts and takes approximately 10 min to complete.^2^ It was emphasized that participating in the study was voluntary and anonymous, and that the collected data would be used only for scientific purposes. An e-mail address was provided if participants had any questions and/or comments about the study.

There were three versions of the text, depending on the narrator’s language: masculine, feminine, and gender neutral. Participants randomly received two texts: a masculine and a gender-neutral text or a feminine and a gender-neutral text. The two texts varied in content, and participants were assigned randomly to version A or B of the text. The texts described everyday activities and short interactions with others (family members or friends). We used everyday activities to ensure that the texts’ protagonists would not be special in any way other than their gender identity, allowing the reader to relate to the texts easily. All participants evaluated gender-neutral texts, part of them (*n* = 61) after a masculine text and part of them (*n* = 75) after a feminine text. Thus, on the crucial comparison between gendered and neutral texts, the study had a within-participant design, and on a secondary comparison between male and female texts, a between-participants design.^3^

For each text, participants were asked whether they were familiar with this kind of language (yes = 1, no = 2). The main dependent variables were then assessed with a few questions measuring text comprehensibility and person evaluation using Likert-type scales, from 1 (*strongly disagree*) to 5 (*strongly agree*). The Text Comprehensibility scale comprised the items understandable, sounds good, and is reliable (*α*_gendered text_ = 0.74, *α*_neutral text_ = 0.64). The Person Evaluation scale comprised a set of four questions asking whether the person from the text seems competent, credible, nice, and whether the participant would like to go out for coffee with this person (*α*_gendered text_ = 0.80, *α*_neutral text_ = 0.76). As a separate question, we included an item from the social distance scale that measures acceptance of a minority member in different spheres that are closer to or more distant from the participant. We chose the most intimate item, asking whether participants would accept a relationship between a person from their family and the person from the text (Bogardus, 1926; Stefaniak et al., 2017). This also was answered on a 1–5 scale, with answers ranging from 1 (*I definitely would be against it*) to 5 (*I definitely would accept it*).

For each text, participants answered the open-ended question What name would you give to the person from the text? The answers were coded accordingly: masculine name = 0, feminine name = 1, and gender-neutral name = 2. Participants also answered three open-ended questions about imaginary scenarios in which they wrote what they would say to the person from the text. In the first scenario, the person from the text was walking in front of the participant, and something fell out of their pocket. The participant picked it up and wanted to address the person from the text. In the second scenario, the participant was working in a bar and thought that the person from the text wanted to order something and wanted to ask this person whether they want to order. In the third scenario, the participant was sitting in a bus full of people, and the person from the text was standing next to them on crutches. The participant wanted to offer them a seat. How the participant addressed the person from the text was coded based on whether they used masculine/feminine or gender-neutral language (masculine = 0, feminine = 1, gender neutral = 2).

After text and person evaluation, we measured contact with non-heteronormative individuals.^4^ The scale comprised six questions (Stefaniak et al., 2017). The first one asked how many non-heteronormative people the participant knows personally. The second one asked how many non-heteronormative individuals the participant’s close ones knew. The third one asked about the number of non-heteronormative friends the participant had. The same questions were asked in terms of knowing gays and/or lesbians. Possible answers were *hard to say* (coded as 1, not used in statistics), 0 people = 2, 1–2 people = 3, a few = 4, many = 5, and very many = 6. As all six items showed high reliability of the scale (*α* = 0.83), the mean of all items (omitting hard to say value) was taken. Finally, participants answered demographic questions about their gender identification with an open-ended question, age, education, and place of residence.

The study’s purpose then was explained to the participants, who were informed that the questionnaire measured reactions to gender-neutral language used by non-binary individuals. There was also a request to share the questionnaire with others but without revealing the study’s purpose. The participants also were invited to leave comments about the study.

## Results

### Familiarity, Names, and Scenarios

Most participants (89%) declared that they were familiar with the language used in the gendered texts. However, the opposite was the case with the gender-neutral texts, in which 81% answered that they were not familiar with this kind of language.

**Fig. 1:**
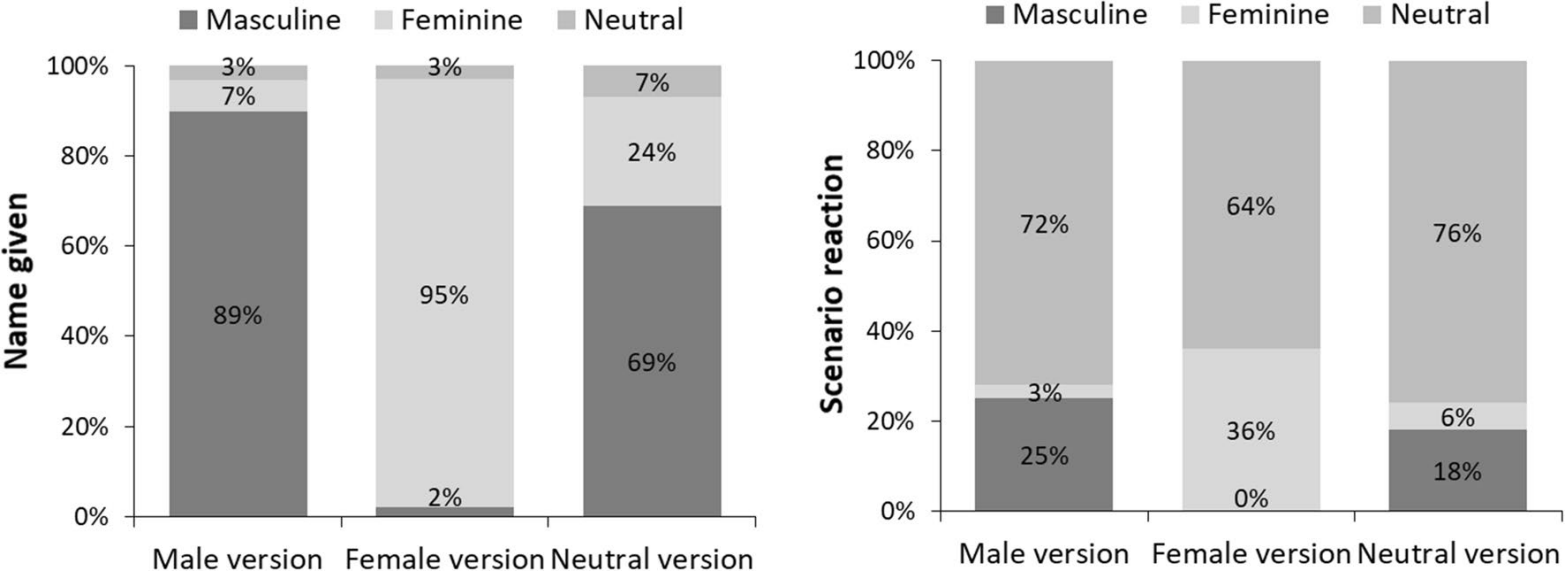
Names given and reactions to scenarios depending on the version of the text

When asked to provide a name for the narrator of the text, for the masculine text, most participants gave a masculine name, and for the feminine text, most gave a feminine name (Fig. 1, left). With the gender-neutral text, most participants gave the person a masculine name, one-fourth a feminine name, and only 7% a gender-neutral name (e.g., *Alex*).

As for the three scenarios, most participants addressed the person from the text in a gender-neutral way, mainly by using *you* or plural and passive forms that avoid gender (Fig. 1, right). This also was the case for masculine and feminine texts but was the most prevalent for the gender-neutral texts. Most of the remaining participants addressed the person from the feminine text in a feminine way and from the masculine text in a masculine way. For the gender-neutral text, just as in the case of name-giving, more participants addressed the person in a masculine than in a feminine way.

### Preliminary Analyses

To verify whether we needed to include covariates in the analyses of variance (ANOVAs), we explored the potential influence of the text version and the participants’ gender, age, education, and place of residence on the dependent variables of text comprehensibility, person evaluation, and social acceptance.

Three 2 (Text Gender: Gendered vs. Neutral) × 2 (Gendered Text: Masculine vs. Feminine) × 2 (Text Version: A vs. B) mixed ANOVAs showed that reading version A or B of the text did not exert significant influence on any of the dependent variables: text comprehensibility, person evaluation, or social acceptance. There were no significant main effects of the text version (*Fs* <  .58*, ps* > .45, $\eta_{p}^{2}$*s* < .004) nor were there any interactions (*Fs* < 1.31*, ps* > .26, $\eta_{p}^{2}$*s* <  .01). Therefore, we combined the data from both versions.

To explore gender differences, we conducted three 2 (Text Gender: Gendered vs. Neutral) × 2 (Gendered Text: Masculine vs. Feminine) × 2 (Participant’s Gender: Woman vs. Man) ANOVAs. The results showed that there was a weak main effect of gender on comprehensibility (*F* = 5.17, *p* = .03, $\eta_{p}^{2}$ = .04): Men evaluated all texts, regardless of how they were written, as more comprehensible (*M* = 3.57, *SD* = 1.05) than women did (*M* = 3.20, *SD* = 0.99). There was no such effect on person evaluation (*F* = 0.21, *p* = .65, $\eta_{p}^{2}$ = .002) or social acceptance (*F* = 0.09, *p* = .77, $\eta_{p}^{2}$ =  .001). As for comprehensibility, there was also a weak three-way interaction (*p* = .02, $\eta_{p}^{2}$ = .04), which boiled down to men evaluating the masculine text as being slightly more comprehensible than women did (*p* = .05, $\eta_{p}^{2}$ =  .03). However, the crucial comparison for our study between neutral and gendered texts was similar for men and women: The gender-neutral text always was evaluated as less comprehensible than the gendered text. On person evaluation and social acceptance, there were no interaction effect, including gender (*Fs* <  .61*, ps* > .44, $\eta_{p}^{2}$*s* <  .005). To sum up, in the current sample, there were very few gender effects that did not replicate on different variables and did not involve the study’s main comparison between gendered and neutral texts.

Correlations showed no significant relationships concerning age, education, and place of residence with comprehensibility, person evaluation, and social acceptance (*rs* < .15, *ps* > .08).

### Text Comprehensibility, Person Evaluation, and Social Acceptance

**Fig. 2:**
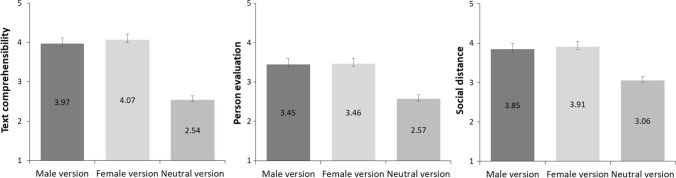
Effect of gendered (masculine, feminine) vs. gender-neutral versions of the text on text comprehensibility (left) and person evaluation (center). Error bars show standard errors of the mean

To compare the effect of a gendered (masculine/feminine) vs. neutral version of the text on text comprehensibility, we conducted a 2 (Text Gender: Gendered vs. Neutral) × 2 (Gendered Text: Masculine vs. Feminine) ANOVA. There was a main effect of gendered vs. neutral text, *F*(1, 134) = 165.51, *p* < .001, $\eta_{p}^{2}$ =  .55, Cohen’s *d* = 1.08, which showed that the participants rated gendered texts as being much more comprehensible (*M* = 4.04, *SD* = 1.16) than the neutral texts (*M* = 2.54, *SD* = 0.86; Fig. 2, left). The main effect of masculine vs. feminine text was not significant, *F*(1, 134) = 0.003, *p* = .96, $\eta_{p}^{2}$ <  .001.

A similar ANOVA for person evaluation showed, again, a large main effect of gendered vs. neutral text, *F*(1, 134) = 121.28, *p* < .001, $\eta_{p}^{2}$ = .48, Cohen’s *d* = 0.95. The participants rated men and women more favorably on the person evaluation scale (*M* = 3.46, *SD* = 0.87) than non-binary individuals from the neutral texts (*M* = 2.57, *SD* = 0.74; Fig. 2, middle). The main effect of masculine vs. feminine text, again, was not significant, *F*(1, 134) = 0.29, *p* = .59, $\eta_{p}^{2}$ = .002.

The last ANOVA was for social acceptance, and it showed, yet again, a large main effect of gendered vs. neutral text, *F*(1, 134) = 86.45, *p* < .001, $\eta_{p}^{2}$ =  .39, Cohen’s *d* = 0.81.^5^ The participants were more keen to accept a man or a woman (*M* = 3.88, *SD* = 0.85) than a non-binary individual as a new member of their family (*M* = 3.06, *SD* = 0.98; Fig. 2, right). The main effect of masculine vs. feminine text was, again, not significant, *F*(1, 134) = 0.004, *p* = .95, $\eta_{p}^{2}$ <  .001.

### Correlations

In terms of contact with non-heteronormative people, our sample had more such contact than an average Pole. Most participants (69%) knew at least one non-heteronormative person, with relatively many knowing a few (36%), and also relatively many (21%) stating that it is hard to say. Most participants (94%) personally knew gays and/or lesbians, with almost half (46%) knowing a few and 4% indicating that it is hard to say.

To explore relationships between the amount of contact with non-heteronormative people and the main dependent measures, correlations were computed (see Table 1). The more non-heteronormative individuals the participants knew, the higher they rated the gender-neutral text and the non-binary individuals from the text. Furthermore, the correlations among the dependent variables showed that the higher the participants rated the gender-neutral text’s comprehensibility, the higher they evaluated the person from the text. The higher the participants evaluated the person speaking in the text, the more willing they were to accept a relationship between a family member and the person from the text.

**Table 1:** Correlations between dependent variables and contact with non-heteronormative people

	Neutral text comprehensibility	Neutral text person evaluation	Neutral text social acceptance	Contact non-heteronormative
Neutral text comprehensibility		0.54***	0.21*	0.18*
Neutral text person evaluation			0.48***	0.19*
Neutral text social acceptance				0.07

* *p* < .05, ** *p* < .01, *** *p* < .001

### Mediation

The ANOVAs showed an effect of the non-binary language on text comprehensibility and on the person evaluation. Furthermore, the correlations showed a relationship between comprehensibility and evaluation. To explore whether the participants’ negative evaluations were due to low text comprehensibility, we tested a mediation model with gendered vs. gender-neutral text as the independent variable, comprehensibility as a mediator, and person evaluation as a dependent variable (Model 4 in Hayes, 2013). We also explored whether contact with non-heteronormative people could moderate these relationships by conducting a moderated mediation (Model 8 in Hayes, 2013). For both analyses, we used 95% bias-corrected bootstrapped confidence intervals based on 5000 bootstrap samples.

**Fig. 3:**
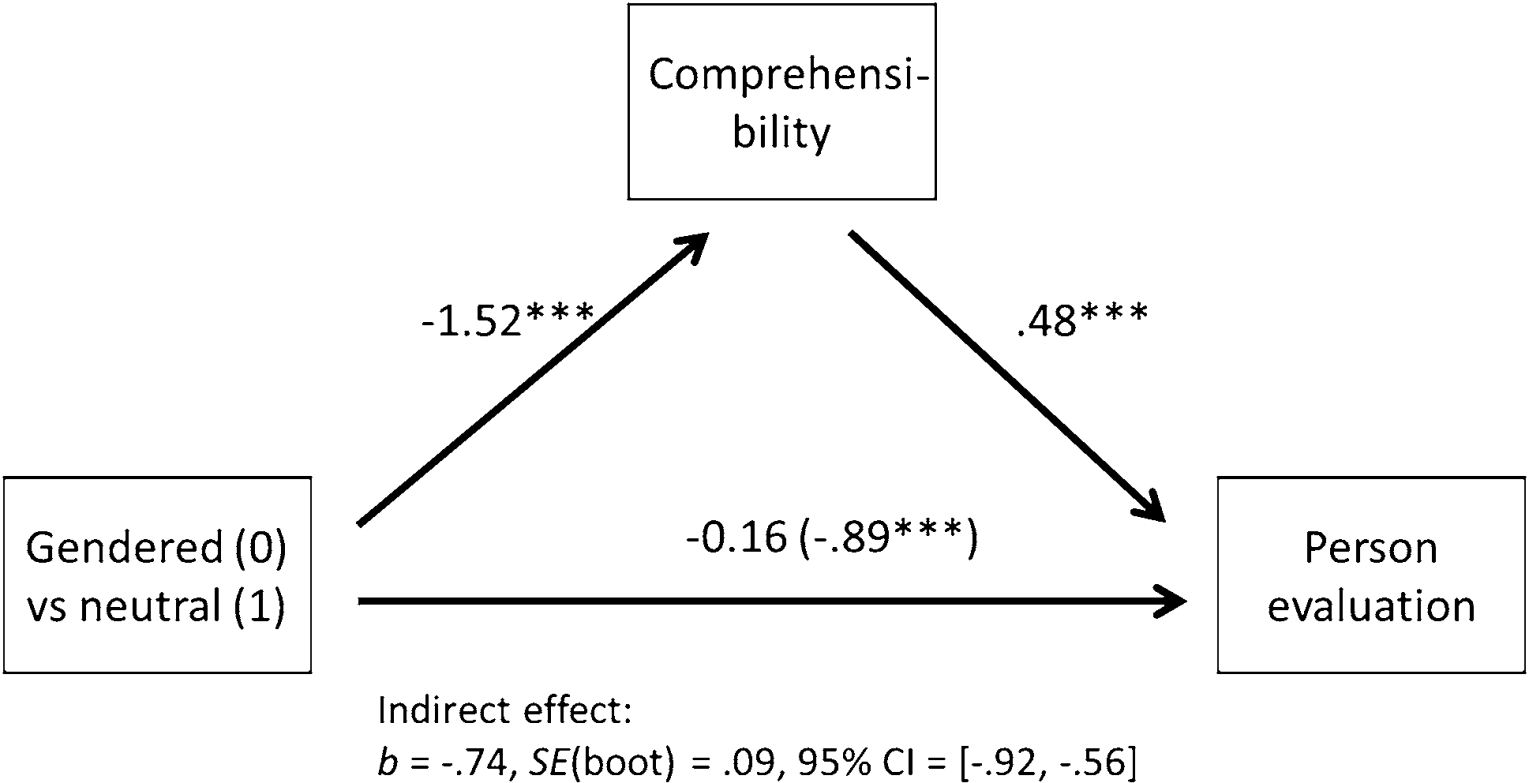
Indirect effect of gendered and neutral texts on person evaluation via comprehensibility

The mediation analysis showed that, as in the earlier ANOVA, people from gender-neutral texts were evaluated much less favorably than people from gendered texts. However, when including comprehensibility, the effect disappeared (Fig. 3). The moderated mediation analysis showed that the influence of the gender-neutral text on comprehensibility was not moderated by contact, *b* = − 0.20, *SE*(boot) = 0.15, *p* = .19, nor was the influence of the text on person evaluation, *b* = 0.04, *SE*(boot) = 0.10, *p* = .66.

## Discussion

The current research shows that non-binary language forms, such as passive voice, are unfamiliar to most listeners or readers and are perceived as less comprehensible than gendered and active language. Furthermore, non-binary people using such language are evaluated more negatively and are socially less accepted than women and men. The negative evaluation could be due to the lower comprehensibility of the language that some non-binary people use. When study participants were asked to give a name to a non-binary person on the basis of their speech, they more often chose a masculine over a feminine name, but when given more freedom on how to address a non-binary person, they often chose gender-neutral language forms, such as *you*.

There may be different reasons for the negative evaluation of non-binary people and their language. First, gender-neutral language can indicate the speaker’s non-normative gender identity. Second, it also may be that participants evaluated non-binary people negatively partly because passive voice is used more frequently to talk about negative emotions and events, so these people may expect more negativity from a person using it (Kałkus, 2015). Third, when it comes to the text itself, our results show that the participants were not accustomed to gender-neutral language. Thus, such language might require more attention, and people using it might suffer from the low-fluency consequences that translate into negative evaluations (Alter & Oppenheimer, 2009). The results of the mediation analysis suggest that the negative evaluation could be explained by low text comprehensibility. However, this result should be approached cautiously because we asked for an evaluation of the person from the text directly after inquiring about text comprehensibility, so these variables can share some measurement variance and make the mediation effect artificially stronger.

If negative evaluation of non-binary people was indeed largely due to the incomprehensibility of their language, familiarizing people with non-binary language could help the social perception of non-binary individuals. Extant research on gender-fair language shows that more exposure to, and preferably usage of, gender-fair language makes people more tolerant not only to such language, but also to the people it describes (Hansen et al., 2016; Koeser et al., 2015). Furthermore, even using gender-neutral language that was designed to help in the visibility of women can also promote more tolerance to LGBT individuals (Tavits & Perez, 2019).

Our results also showed that more contact with non-heteronormative individuals was related to better evaluation of the non-binary text and the person from the text. However, the amount of contact did not moderate the relationship between low comprehensibility and negative evaluation. It seems that both people who have less as well as people who have more contact with non-heteronormative individuals tend to evaluate non-binary people worse if they perceived their language as incomprehensible.

As expected, most people gave the non-binary person a masculine name, which is, regrettably, in line with the male-as-the-norm phenomenon and vast research showing that people assume that the protagonist or the narrator of a text is male even when described using gender-neutral language (see reviews by Sczesny et al., 2016; Stahlberg et al., 2007). Our questionnaire also included exemplary situations that involved meeting a non-binary person and asked participants to imagine how they would address this person. Most participants addressed the unknown person in a gender-neutral way. Participants often did so by using *you,* which is very direct and slightly impolite in Polish. However, it is one way that some non-binary people prefer to be addressed (Petriczko, 2015). The fact that the participants often gave a non-binary person a masculine name but addressed them in a gender-neutral way, can be explained in two ways at the same time. First, participants used what the language allowed them. Virtually all names in Polish are either masculine or feminine, and only a few participants knew foreign names that do not reveal gender (e.g., Alex) or were creative in inventing names. When it came to addressing a non-binary person without using a name, it was easier for participants to use neutral language. Second, one could expect that people would be using *you* form in the case of the gender-neutral text only, but a similar, even if slightly less pronounced, result occurred across all the texts. It could be that the sample recruited influenced the results. Probably a sample comprised of an older generation of participants would rather stick to the politeness norm of addressing individuals as Sir/Madam (*Pan/Pani*) and would, thus, choose a gendered form. It could also be that if the people in the scenarios were described as much older than an average participant, even younger participants might tend to use Sir/Madam.

Based on the current results, we can say that there is a chance that the participants would communicate with non-binary individuals in a way that the latter prefer. Of course, based on these responses, we have hope, but not certainty, as to what kind of language our participants would use if they wanted to engage in a full conversation with a non-binary person. It is possible that in a longer conversation, they would make assumptions about a non-binary person’s gender based on their appearance and would decide on the use of masculine or feminine linguistic forms (Wong, 2017).

Future research could also ask other questions. We decided to ask how would the participant addresses a non-binary person to get their attention, but one could first ask Would you help this person? Whereas our question was constructed to tell us something about the language participants would use (already assuming they want to get the person’s attention), a question about readiness to help would tell us something about the potential discrimination.

### Strengths and Limitations

As in all research with non-representative samples, the current results should not be generalized to all languages, cultures, or to the whole Polish population. Our participants were more likely than an average Pole to be female, live in bigger cities, and were relatively young. Most also had contact with non-heteronormative individuals, which does not reflect the low amount of contact in the whole Polish population (Stefaniak et al., 2017). This all was probably due to the fact that the data were collected using a snowball method, and the questionnaire was posted on a private social media account. However, considering this sample composition, the negative evaluations and big differences between gendered and neutral texts are even more striking. A study with a sample more representative of the Polish population probably would show even more negativity.

A strength of the study when it comes to the sample is that we chose a powerful within-participant design. Regrettably for non-binary people, we also observed statistically large effects. Both are rare in social psychology, but they gave our study more statistical power.

As mentioned, participants were asked to judge the person based only on their language, but we did not take into account their appearance or voice (Hansen et al., 2017). However, research on thin slices of behavior shows that even when examining much less than what we provided might be enough to form a long-lasting impression of a person (Ambady et al., 2000).

### Conclusion

The negative evaluations of non-binary people in our study are alarming. It seems that language that non-binary people use can contribute to these negative perceptions. Even if this one study cannot answer many questions, we hope that it will pave an avenue for more research on the perception of non-binary people and their language. Future research could, for example, focus on other aspects of non-binary individuals’ language. Furthermore, to improve attitudes toward non-binary people, educational programs could talk about who non-binary people are, what kind of language they may use, and how they prefer to be addressed.

## Supplementary Information

Below is the link to the electronic supplementary material.

Supplementary file1 (DOCX 27 kb)

Supplementary file2 (SAV 826 kb)

Supplementary file3 (DOCX 29 kb)

## Footnotes

1 On some measures that were assessed before demographics, there were more answers. To value all participants’ time, we used as many answers as possible for each variable. As a consequence, the *dfs* vary for different analyses, but the *N* is never lower than 130.

2 For all details of the measures used, see the questionnaire in Polish and English in the supplementary materials.

3 As we could not locate a similar study, it was hard to have an expectation to the studied differences’ effect sizes. We chose a within-subject design on our crucial comparison, as such designs are very efficient in terms of statistical power and require small samples. Assuming a medium effect size (Cohen’s *d* = 0.50) for the comparison of the gendered and neutral texts on our main dependent variables, we needed an *N* of 45 to attain a power of 0.95. This was achieved quickly, and we let the study run a bit longer.

4 We are aware of the fact that asking for contact with specifically non-binary individuals would have been a more accurate measure of contact, but we suspected that some participants would not know this term and most participants would indicate that they do not know anyone. To avoid such a floor effect, we decided to ask for contact with non-heteronormative people as a useful proxy.

5 The detected effects were large. For such effects, a necessary sample size (with α = .05 and power = .95) would need to be between 11 and 18 participants. Our study had an achieved power of 1.
