# Supplementary material for: Social Perception of Non-Binary Individuals
Source: Arch Sex Behav. 2022 Apr 25;51(4):2027–35. doi: 10.1007/s10508-021-02234-y (PMC9192499; doi:10.1007/s10508-021-02234-y)
Supplement: Supplementary file 1 — Supplementary file1 (DOCX 27 kb) [file 10508_2021_2234_MOESM1_ESM.docx]

Dzień dobry,

jestem studentką trzeciego roku Warsaw International Studies in Psychology na Uniwersytecie Warszawskim, pracującą nad swoją pracą empiryczną. Ten kwestionariusz bada relację między językiem a emocjami, co jest tematem mojej pracy. Wypełnienie kwestionariusza zajmuje około 10 minut i składa się z trzech części. Dwie pierwsze części polegają na przeczytaniu tekstu i odpowiedzeniu na pytania z nim związane. Trzecią częścią są pytania demograficzne. Wypełnienie kwestionariusza jest dobrowolne i anonimowe. Zebrane dane zostaną wykorzystane tylko do pracy nad badaniem. W przypadku dodatkowych pytań dotyczących badania i kwestionariusza można się ze mną skontaktować wysyłając maila na adres: k.zoltak@student.uw.edu.pl

Dziękuję za wypełnienie ankiety,

Katarzyna Żółtak

=================================================

Przed Tobą pierwszy tekst. Przeczytaj go i odpowiedz na pytania jak najbardziej szczerze i dokładnie jak możesz.

**Tekst A, wersja żeńska**

Siedziałam_fem_ ze znajomymi. Co dzisiaj robiłaś_fem_? - usłyszałam_fem_. Jak zwykle, do pracy miałam_fem_ na 8, więc wstałam_fem_ o 7, żeby się wyszykować i wyprowadzić psa na spacer. Nie było czasu na wypicie kawy, więc chciało mi się potem spać. Po pracy chciałam_fem_ jeszcze iść na basen, ale jestem trochę przeziębiona_fem_, więc jednak zrezygnowałam_fem_. Na obiad zjadłam_fem_ makaron ze szpinakiem i znowu wyszłam_fem_ z psem. Potem musiałam_fem_ iść na autobus, żeby się z Wami spotkać. Bardzo fajnie Was widzieć.

**Tekst B, wersja żeńska**

Byłam_fem_ w sklepie. Usłyszałam_fem_, że mój telefon zadzwonił. To było moje rodzeństwo z którymi umówiłam_fem_ się na spotkanie tego samego dnia po południu. Po rozmowie skończyłam_fem_ zakupy, wróciłam_fem_ do domu i zaczęłam_fem_ robić obiad. Zjadłam_fem_ obiad ze smakiem i nie mogłam_fem_ się już doczekać wyjścia z domu, bo była ładna pogoda. Przez pogodę zamiast jechać tramwajem poszłam_fem_ na spotkanie na piechotę.

**Tekst A, wersja męska**

Siedziałem_mas_ ze znajomymi. Co dzisiaj robiłeś_mas_? – usłyszałem_mas_. Jak zwykle, do pracy miałem_mas_ na 8, więc wstałem_mas_ o 7, żeby się wyszykować i wyprowadzić psa na spacer. Nie było czasu na wypicie kawy, więc chciało mi się potem spać. Po pracy chciałem_mas_ jeszcze iść na basen, ale jestem trochę przeziębiony, więc jednak zrezygnowałem_mas_. Na obiad zjadłem_mas_ makaron ze szpinakiem i znowu wyszedłem_mas_ z psem. Potem musiałem_mas_ iść na autobus, żeby się z Wami spotkać. Bardzo fajnie Was widzieć.

**Tekst B, wersja męska**

Byłem_mas_ w sklepie. Usłyszałem_mas_, że mój telefon zadzwonił. To było moje rodzeństwo z którymi umówiłem_mas_ się na spotkanie tego samego dnia po południu. Po rozmowie skończyłem_mas_ zakupy, wróciłem_mas_ do domu i zacząłem_mas_ robić obiad. Zjadłem_mas_ obiad ze smakiem i nie mogłem_mas_ się już doczekać wyjścia z domu, bo była ładna pogoda. Przez pogodę zamiast jechać tramwajem poszedłem_mas_ na spotkanie na piechotę.

Q1. Czy spotkałeś/aś się wcześniej z takim językiem?

- Tak (1)
- Nie (2)

Q2. Zaznacz w jakim stopniu zgadzasz się z poniższymi stwierdzeniami.

|  | Zdecydowanie się nie zgadzam | Raczej się nie zgadzam | Ani się zgadzam ani się nie zgadzam | Raczej się zgadzam | Zdecydowanie się zgadzam |
| --- | --- | --- | --- | --- | --- |
| Ten tekst jest zrozumiały |  |  |  |  |  |
| Ten tekst dobrze brzmi |  |  |  |  |  |
| Ten tekst jest wiarygodny |  |  |  |  |  |

Q3. Zaznacz w jakim stopniu zgadzasz się z poniższymi stwierdzeniami.

|  | Zdecydowanie się nie zgadzam | Raczej się nie zgadzam | Ani się zgadzam ani się nie zgadzam | Raczej się zgadzam | Zdecydowanie się zgadzam |
| --- | --- | --- | --- | --- | --- |
| Czy osoba z tekstu wydaje Ci się kompetentna? |  |  |  |  |  |
| Czy osoba z tekstu wydaje Ci się wiarygodna? |  |  |  |  |  |
| Czy osoba z tekstu wydaje Ci się sympatyczna? |  |  |  |  |  |
| Czy chciał(a)byś wyjść z osobą z tekstu na kawę? |  |  |  |  |  |

Q4. Odpowiedz na pytanie.

|  | Zdecydowanie był(a)bym przeciwny/a | Raczej był(a)bym przeciwny/a | Nie był(a)bym ani przeciwna ani bym zaakceptował/a | Raczej bym zaakceptował/a | Zdecydowanie bym zaakceptował/a |
| --- | --- | --- | --- | --- | --- |
| Czy zaakceptował(a)byś związek członka Twojej rodziny z osobą z tekstu? |  |  |  |  |  |

Q5. Jakie imię nadał(a)byś osobie z tekstu?

________________________________________________________________

Przeczytaj opisy sytuacji i odpowiedz na pytania.

Q6. Wyobraź sobie, że osoba z tekstu idzie przed Tobą chodnikiem i wypada jej coś z kieszeni. Podnosisz to i chcesz zwrócić uwagę tej osoby. Co powiesz w tej sytuacji?

________________________________________________________________

Q7. Wyobraź sobie, że pracujesz w barze. Osoba z tekstu stoi przy barze i wydaje Ci się, że chce coś zamówić. Masz zamiar zapytać się jej o to, czy coś jej podać. Co powiesz w tej sytuacji?

________________________________________________________________

Q8. Wyobraź sobie, że siedzisz w zatłoczonym autobusie. Osoba z tekstu stoi obok Ciebie i jest o kulach. Chcesz ustąpić jej miejsca. Co powiesz w tej sytuacji?

____________________________________________________________

==================================================

Przed Tobą drugi tekst. Przeczytaj go i odpowiedz na pytania jak najbardziej szczerze i dokładnie, jak możesz.

**Tekst A, wersja neutralna**

Siedziało_neu_ mi się ze znajomymi. Co Ci się dzisiaj robiło_neu_? – usłyszało_neu_ mi się. Jak zwykle, do pracy miało_neu_ mi się na 8, więc wstało_neu_ mi się o 7, żeby się wyszykować i wyprowadzić psa na spacer. Nie było czasu na wypicie kawy, więc chciało_neu_ mi się potem spać. Po pracy chciało_neu_ mi się jeszcze iść na basen, ale przeziębiło_neu_ mi się, więc jednak mi się zrezygnowało_neu_. Na obiad zjadło_neu_ mi się makaron ze szpinakiem i znowu wyszło_neu_ mi się z psem. Potem musiało_neu_ mi się iść na autobus, żeby się z Wami spotkać. Bardzo fajnie Was widzieć.

**Tekst B, wersja neutralna**

Było_neu_ mi się w sklepie. Usłyszało_neu_ mi się, że mój telefon zadzwonił. To było moje rodzeństwo z którymi umówiło_neu_ mi się na spotkanie tego samego dnia po południu. Po rozmowie skończyło_neu_ mi się zakupy, wróciło_neu_ do domu i zaczęło_neu_ mi się robić obiad. Obiad zjadło_neu_ mi się ze smakiem i nie mogło_neu_ mi się już doczekać wyjścia z domu, bo była ładna pogoda. Przez pogodę zamiast jechać tramwajem poszło_neu_ mi się na spotkanie na piechotę.

[Q1 (teraz Q9) do Q8 (Q16) znów.]

==================================================

Q17. Odpowiedz na pytania.

|  | Trudno powiedzieć | 0 osób | 1-2 osoby | Kilka | Wiele | Bardzo wiele |
| --- | --- | --- | --- | --- | --- | --- |
| Ile znasz osobiście osób nieheteronormatywnych? |  |  |  |  |  |  |
| Ile znasz osobiście gejów lub lesbijek? |  |  |  |  |  |  |
| Ile Twoi bliscy znają osób nieheteronormatywnych? |  |  |  |  |  |  |
| Ile Twoi bliscy znają gejów lub lesbijek? |  |  |  |  |  |  |
| Ile masz wśród przyjaciół osób nieheteronormatywnych? |  |  |  |  |  |  |
| Ile masz wśród przyjaciół gejów lub lesbijek? |  |  |  |  |  |  |

Q18. Płeć

________________________________________________________________

Q19. Wiek (w latach)

________________________________________________________________

Q20. Wykształcenie

- Podstawowe
- Średnie zawodowe
- Średnie maturalne
- W trakcie studiów
- Wyższe

Q21. Miejsce zamieszkania

- Wieś
- Miasto poniżej 50 tys. mieszkańców
- Miasto od 50 tys. mieszkańców do 200 tys. mieszkańców
- Miasto od 200 tys. mieszkańców do 500 tys. mieszkańców
- Miasto powyżej 500 tys. mieszkańców

==================================================

To już koniec badania. Dziękuję za wzięcie w nim udziału.

Celem badania było zbadanie, jakie uczucia wywołuje język neutralny płciowo, którego używają na co dzień osoby niebinarne. Zachęcam do zapraszania znajomych do wypełnienia ankiety. Jeśli to zrobisz, nie informuj ich jednak o celu badania aż do momentu wypełniania przez nich kwestionariusza.

Jeśli masz jakieś komentarze dotyczące badania napisz je tutaj. Jeśli nie, zakończ badanie.

________________________________________________________________
