# Supplementary material for: Social Perception of Non-Binary Individuals
Source: Arch Sex Behav. 2022 Apr 25;51(4):2027–35. doi: 10.1007/s10508-021-02234-y (PMC9192499; doi:10.1007/s10508-021-02234-y)
Supplement: Supplementary file 3 — Supplementary file3 (DOCX 29 kb) [file 10508_2021_2234_MOESM3_ESM.docx]

Hello,

I am a third-year student of Warsaw International Studies in Psychology at the University of Warsaw, and I am working on my empirical thesis. This questionnaire examines the relationship between language and emotions, which is the subject of my thesis. The questionnaire takes about 10 minutes to complete and has three parts. The first two parts comprise reading a text and answering questions related to it. The third part contains demographic questions. Completing the questionnaire is voluntary and anonymous. Collected data will be used only for the present study’s purposes. If you have any questions about the study and the questionnaire, you can contact me at: k.zoltak@student.uw.edu.pl

Thank you for participating in the study,

Katarzyna Żółtak

==================================================

Here is the first text. Read it and answer the questions as honestly and accurately as you can.

[One out of the four texts is presented.]

**Text A, feminine version**

I was sitting_fem_ with my friends. What did you do_fem_ today? - I heard_fem_.

As usual, I had_fem_ to start working at 8 a.m., so I got_fem_ up at 7 a.m. to get ready and take the dog for a walk. There was no time to drink coffee, so I was sleepy later on. After work, I wanted_fem_ to go to the swimming pool, but I’ve got a little cold_fem_, so I decided_fem_ not to go. For dinner, I ate_fem_ pasta with spinach, and I went_fem_ out with the dog again. Then I had_fem_ to go to the bus to meet you. It’s great to see you.

**Text B, feminine version**

I was_fem_ in a store. I’ve heard_fem_ my phone ringing. It was my siblings, with whom I made_fem_ an appointment for the same day in the afternoon. After the conversation, I finished_fem_ shopping, returned_fem_ home, and started_fem_ making dinner. I ate_fem_ pleasurably and couldn’t_fem_ wait to leave the house because the weather was so nice. Because of the weather, instead of taking the tram, I walked_fem_ to the meeting.

**Text A, masculine version**

I was sitting_masc_ with my friends. What did you do_masc_ today? - I heard_masc_.

As usual, I had_masc_ to start working at 8 a.m., so I got_masc_ up at 7 a.m. to get ready and take the dog for a walk. There was no time to drink coffee, so I was sleepy later on. After work, I wanted_masc_ to go to the swimming pool, but I’ve got a little cold_masc_, so I decided_masc_ not to go. For dinner, I ate_masc_ pasta with spinach, and I went_masc_ out with the dog again. Then I had_masc_ to go to the bus to meet you. It’s great to see you.

**Text B, masculine version**

I was_masc_ in a store. I’ve heard_masc_ my phone ringing. It was my siblings, with whom I made_masc_ an appointment for the same day in the afternoon. After the conversation, I finished_masc_ shopping, returned_masc_ home, and started_masc_ making dinner. I ate_masc_ with pleasure and couldn’t_masc_ wait to leave the house because the weather was so nice. Because of the weather, instead of taking the tram, I walked_masc_ to the meeting.

Q1. Have you encountered such language before?

- Yes
- No

Q2. Please indicate how much you agree with the statements below.

|  | Strongly disagree | Somewhat disagree | Neither agree nor disagree | Somewhat agree | Strongly agree |
| --- | --- | --- | --- | --- | --- |
| The text is understandable |  |  |  |  |  |
| The text sounds good |  |  |  |  |  |
| The text is reliable |  |  |  |  |  |

Q3. Please indicate how much you agree with the statements below.

|  | Strongly disagree | Somewhat disagree | Neither agree nor disagree | Somewhat agree | Strongly agree |
| --- | --- | --- | --- | --- | --- |
| Does the person from the text seem competent to you? |  |  |  |  |  |
| Does the person from the text seem credible to you? |  |  |  |  |  |
| Does the person from the text seem nice to you? |  |  |  |  |  |
| Would you like to meet the person from the text for coffee? |  |  |  |  |  |

Q4. Answer the question.

|  | I definitely would be against it | I likely would be against it | I would be neither against it, nor would I accept it | I likely would accept it | I definitely would accept it |
| --- | --- | --- | --- | --- | --- |
| Would you approve of a relationship between a family member and the person from the text? |  |  |  |  |  |

Q5. What name would you give to the person from the text?

________________________________________________________________

Read the situation descriptions below and answer the questions:

Q6. Imagine that the person from the text is walking ahead of you on a sidewalk and something falls out of their pocket. You pick it up, and you want to get the person’s attention. What would you say in this situation?

________________________________________________________________

Q7. Imagine you’re working in a bar. The person from the text is standing at the bar, and you think they want to order something. You are going to ask them whether they want something. What would you say in this situation?

________________________________________________________________

Q8. Imagine that you are sitting on a crowded bus. The person from the text stands next to you and is on crutches. You want to offer them your seat. What would you say in this situation?

______________________________________________________________

==================================================

Here is the second text. Read it and answer the questions as honestly and accurately as you can.

[One out of the two texts is presented: B if the first text was A, and A if the first text was B.]

**Text A, neutral version**

I was sitting_neutr_passive_ with my friends. What did you do_neutr_passive_ today? - I heard_neutr_passive_.

As usual, I had_neutr_passive_ to start working at 8 a.m., so I got_neutr_passive_ up at 7 a.m. to get ready and take the dog for a walk. There was no time to drink coffee, so I was sleepy later on. After work, I wanted_neutr_passive_ to go to the swimming pool, but I’ve got a little cold_neutr_passive_, so I decided_neutr_passive_ not to go. For dinner, I ate_neutr_passive_ pasta with spinach, and I went_neutr_passive_ out with the dog again. Then I had_neutr_passive_ to go to the bus to meet you. It’s great to see you.

**Text B, neutral version**

I was_neutr_passive_ in a store. I’ve heard_neutr_passive_ my phone ringing. It was my siblings, with whom I made_neutr_passive_ an appointment for the same day in the afternoon. After the conversation, I finished_neutr_passive_ shopping, returned_neutr_passive_ home, and started_neutr_passive_ making dinner. I ate_neutr_passive_ with pleasure and couldn’t_neutr_passive_ wait to leave the house because the weather was so nice. Because of the weather, instead of taking the tram, I walked_neutr_passive_ to the meeting.

[Q1 (now Q9) till Q8 (Q16) again.]

==================================================

Q17. Answer the questions.

|  | Hard to say | 0 people | 1-2 people | A few | Many | Very many |
| --- | --- | --- | --- | --- | --- | --- |
| How many non-heteronormative people do you know personally? |  |  |  |  |  |  |
| How many gays or lesbians do you know personally? |  |  |  |  |  |  |
| How many non-heteronormative people do your close ones know? |  |  |  |  |  |  |
| How many gays or lesbians do your close ones know? |  |  |  |  |  |  |
| How many non-heteronormative friends do you have? |  |  |  |  |  |  |
| How many gay or lesbian friends do you have? |  |  |  |  |  |  |

Q18. Gender

________________________________________________________________

Q19. Age (in years)

________________________________________ ________________________

Q20. Education

- Primary
- Secondary vocational
- Secondary
- During studies
- Higher

Q21. Place of residence

- Countryside
- City/town with fewer than 50,000 residents
- City with 50,000 to 200,000 residents
- City with 200,000 to 500,000 residents
- City with over 500,000 residents

==================================================

This is the end of the study. Thank you for participating.

This study aimed to investigate the perception of gender-neutral language that non-binary people use. I encourage you to invite your friends to complete the survey as well. However, if you do this, do not tell them this study’s purpose until they complete the questionnaire.

If you have any comments about the study, write them here. If not, finish the survey.

________________________________________________________________
